# Icaritin-loaded PLGA nanoparticles activate immunogenic cell death and facilitate tumor recruitment in mice with gastric cancer

**DOI:** 10.1080/10717544.2022.2079769

**Published:** 2022-05-30

**Authors:** Yao Xiao, Wenxia Yao, Mingzhen Lin, Wei Huang, Ben Li, Bin Peng, Qinhai Ma, Xinke Zhou, Min Liang

**Affiliations:** a Department of Oncology, Innovation centre for Advanced Interdisciplinary Medicine, Guangzhou Key Laboratory of Enhanced Recovery after Abdominal Surgery, The Fifth Affiliated Hospital of Guangzhou Medical University, Guangzhou Medical University, Guangzhou, 510700, China; b State Key Laboratory of Respiratory Disease, National Clinical Research Center for Respiratory Disease, Guangzhou Institute of Respiratory Health, the First Affiliated Hospital of Guangzhou Medical University, Guangzhou Medical University, Guangzhou, 510062, China

**Keywords:** Icaritin, poly (lactic-co-glycolic acid) (PLGA), Immunogenic cell death (ICD), Mitochondrial oxidative damage

## Abstract

This study aimed to explore the anti-tumor effect of icaritin loading poly (lactic-co-glycolic acid) nanoparticles (refer to PLGA@Icaritin NPs) on gastric cancer (GC) cells. Transmission Electron Microscope (TEM), size distribution, zeta potential, drug-loading capability, and other physicochemical characteristics of PLGA@Icaritin NPs were carried out. Furthermore, flow cytometry, confocal laser scanning microscope (CLSM), Cell Counting Kit-8 (CCK-8), Transwell, Elisa assay and Balb/c mice were applied to explore the cellular uptake, anti-proliferation, anti-metastasis, immune response activation effects, and related anti-tumor mechanism of PLGA@Icaritin NPs *in vitro* and *in vivo*. PLGA@Icaritin NPs showed spherical shape, with appropriate particle sizes and well drug loading and releasing capacities. Flow cytometry and CLSM results indicated that PLGA@Icaritin could efficiently enter into GC cells. CCK-8 proved that PLGA@Icaritin NPs dramatically suppressed cell growth, induced Lactic dehydrogenase (LDH) leakage, arrested more GC cells at G2 phase, and inhibited the invasion and metastasis of GC cells, compared to free icaritin. In addition, PLGA@Icaritin could help generate dozens of reactive oxygen species (ROS) within GC cells, following by significant mitochondrial membrane potentials (MMPs) loss and excessive production of oxidative-mitochondrial DNA (Ox-mitoDNA). Since that, Ox-mitoDNA further activated the releasing of damage associated molecular pattern molecules (DAMPs), and finally led to immunogenic cell death (ICD). Our *in vivo* data also elaborated that PLGA@Icaritin exerted a powerful inhibitory effect (∼80%), compared to free icaritin (∼60%). Most importantly, our results demonstrated that PLGA@Icaritin could activate the anti-tumor immunity via recruitment of infiltrating CD4+ cells, CD8+ T cells and increased secretion of cytokine immune factors, including interferon-γ (IFN-γ) tumor necrosis factor-α (TNF-α) and interleukin-1 (IL-1).^++^ Our findings validate that the successful design of PLGA@Icaritin, which can effectively active ICD and facilitate tumor recruitment in GC through inducing mitoDNA oxidative damage.

## 1. Introduction

Cancer is a challenging health problem worldwide and reduces life expectancy. There are more than 19 million new cases of cancer diagnosed and 10 million deaths from cancer worldwide each year, of which 1.08 million cases are of gastric cancer (GC), which causes nearly 800,000 deaths every year (Sung et al., 2021). Although the diagnosis and treatment of GC have improved in recent years, its morbidity and mortality rates are increasing and the 5-year survival rate remains very low (Allemani et al., 2018). The uncontrolled proliferation and extensive metastatic abilities of GC cells lead to a high recurrence rate, making treatment even more difficult (Smyth et al., 2020). Chemotherapy is preferred as an adjunct to kill tumor cells; however, chemotherapeutic resistance makes effective systemic therapy challenging in GC and results in tumor recurrence and metastasis (Vasan et al., 2019). Using higher concentrations of drugs or administering drugs at a higher frequency can lead to serious toxic side effects and strong drug resistance (Auberger et al., 2020). Therefore, novel treatments for GC are needed.

Immunogenic cell death (ICD) is a unique physiological form of regulatory cell death that can be induced by chemotherapeutic drugs such as anthracyclines and oxaliplatin (Wang et al., 2018; Ahmed & Tait, 2020). These drugs stimulate cancer cells to release damage-related model molecules, including calreticulin (CRT), adenosine-triphosphate (ATP), high mobility group protein B1 (HMGB1), CXCL1, and CCL2. During ICD, calreticulin is exposed on the cell surface, conveying an ‘eat me’ signal that stimulates dendritic cells (DC). Dying tumor cells release ATP, which is equivalent to the ‘find me’ signal, and helps recruit T cells into the solid tumor, followed by initiation of the immune response (Galluzzi et al., 2020), leading to tumor cell death via positive feedback. For instance, DOX/aNLG919-loaded CaCO nanoparticles can cause ICD in cancer cells and exert persistent anti-tumor effects (Li et al., 2021). Therefore, triggering ICD may overcome clinical problems as additional immune therapy.

Clinical studies showed that Chinese herbal medicines can function as anti-cancer drugs to inhibit tumor cells (Luo et al., 2019) while causing fewer toxic side effects (Xue, 2016). Icaritin can affect a variety of physiological and pathological processes, such as by preventing osteoporosis (Huang et al., 2018) and immunomodulation (Qin et al., 2020) and promoting osteogenic differentiation of bone marrow mesenchymal stem cells (Wu et al., 2017; Wei et al., 2020), and is preferred as an anti-tumor therapy. Interestingly, icaritin enhances the anti-tumor immune response in cancer (Tao et al., 2021). Icaritin can induce mitochondrial damage-related apoptosis, and ICD inducers cause ICD by targeting endoplasmic reticulum stress, mitochondrial oxidative stress, and mitochondrial apoptosis (Jin et al., 2017; Chen et al., 2019; Li et al., 2020). Based on these studies, we predicted that icaritin could activate ICD by inducing mitochondrial oxidative stress. However, the poor internal penetration and transporter-mediated efflux of icaritin lead to low bioavailability, limits its clinical application (Han et al., 2019).

Nanomedicine is useful for tumor treatment (Adiseshaiah et al., 2016; Cruz & Kayser, 2019; Liu et al., 2019) and can improve treatment effects, reduce side effects, and overcome drug resistance. Poly lactic-co-glycolic acid (PLGA) is a biodegradable material that can be hydrolyzed into the biodegradable metabolites lactic acid and glycolic acid (Li & Jiang, 2018). PLGA is a suitable nanodrug carrier because of its biodegradability, small size, and high biocompatibility. Polyethylene glycol (PEG) can modify the surface of PLGA, increasing the blood circulation half-life of PLGA (Duan et al., 2021). Therefore, PLGA nanoparticles (NPs) are particularly attractive for clinical application as drug delivery systems. For example, nanocarriers composed of PLGA carrying metformin increase the anticancer effects of metformin on ovarian cancer cells (Faramarzi et al., 2019), and PLGA particles enhance the immunotherapy effect of DC-based vaccines (Allahyari & Mohit, 2016). However, the inhibitory effect, ICD activation efficiency, and related mechanism of PLGA@Icaritin in GC cells remain unclear. In this study, we constructed icaritin-loaded PLGA NPs; examined their physical and chemical properties, such as the particle size, zeta potential, and drug-loaded capacity; and evaluated the cellular uptake, anti-proliferation, anti-metastasis, and immune response activation effects and related anti-tumor mechanisms of PLGA@Icaritin NPs *in vitro* and *in vivo*.

## 2. Materials and methods

### 2.1. Materials and characterization

#### 2.1.1. Chemicals

Poly (D, _L_-lactide-co-glycolide) (PLGA), *N*-hydroxysuccinimide, 1-ethyl-3-(3-dimethly-aminopropyl) carbodiimide, dichloromethane, icaritin, 4-(dimethylamino) pyridine, 3β-[*N*′-(*N*′,*N*′-dimethylaminoethane)-carbamoyl] cholesterol hydrochloride (DC-cholesterol HCl), and chloroform were purchased from Sigma (St. Louis, MO, USA). PEG (NH_2_-mPEG-NH_2_, MW 2000, 98%) was obtained from Guangzhou Tanshui Co., Ltd. (Guangzhou, China). All other chemicals were of extra-pure grade.

#### 2.1.2. Animals

Male BALB/c mice, aged 5–7 weeks, were purchased from the Experimental Animal Center of Guangzhou Medical University, which is a facility certified by the Guangdong Provincial Bureau of Science. Animal experiments were performed according to ethical guidelines, and relevant national authorities permitted these experiments.

### 2.2. Synthesis of PLGA@iicaritin

PLGA polymers were synthesized as previously reported (Tabatabaei et al., 2016). PLGA-PEG copolymers (PEG2000) were prepared by melt polymerization under vacuum using stannous octoate [stannous 2-ethylhexanoate] as a catalyst. DL-Lactide (1.441 g), glycolide (0.285 g), and PEG2000 (0.77 g) (45%/w) were heated to 140 °C in a bottleneck flask under a nitrogen atmosphere for complete melting. The molar ratio of DL-lactide to glycolide was 3:1. Stannous octoate [0.05% (w/w)] was added, and the temperature of the reaction mixture was increased to 180 °C. This temperature was maintained for 4 h. Polymerization was performed under vacuum. The copolymer was recovered by dissolution in methylene chloride, followed by precipitation in ice-cold diethyl ether. After 24 h, PLGA-PEG was purified by washing with ethanol and drying under vacuum. Icaritin (0.5 mg) was dissolved in 500 μL dimethyl sulfoxide and mixed with 5 mg of PLGA-PEG in the drug solution. The drug-polymer mixture was added dropwise to 10 mL of deionized water while stirring and stirred for 24 h at room temperature in a beaker. PLGA@Icaritin was obtained, dialyzed to remove the organic solvent, and freeze-dried.

### 2.3. Physicochemical characterization

A Hitachi HT7700 transmission electron microscope (Tokyo, Japan) was used to detect the NPs. A ZetaPALS system (Brookhaven Instruments, Holtsville, NY) was used to detect the average size and surface potential of the synthesized NPs. Fourier transform infrared (FTIR) spectra were obtained using a Nicolet/Nexus 670 FTIR analyzer (Thermo Fisher Scientific, Waltham, MA, USA) at frequencies of 500–4000 cm^−1^.

### 2.4. *In vitro* drug release curve

Icaritin and PLGA co-loaded nanoparticle solution (2 mL) in dialysis bags (Thermo Fisher Scientific, MWCO = 10 kDa) were incubated in release medium [100 mL, 0.01 M phosphate-buffered saline (PBS) containing 1% bovine serum albumin)] for 24 h at 37 °C. At different time points, 0.1 mL of the sample was withdrawn and replaced with the same volume of blank solution. The contents of icaritin and PLGA in the collected media were quantified using HPLC (Shimadzu, Kyoto, Japan) (UV detection at 270 nm, 0.1% phosphoric acid, and mobile phase of acetonitrile (55:45) for icaritin; UV detection at 262 nm, 0.1% phosphoric acid, and mobile phase of acetonitrile (75:25) for PLGA).

### 2.5. Cell culture

Dulbecco’s modified Eagle’s medium high glucose (DMEM-HG), fetal bovine serum (FBS), and trypsin-EDTA (0.05%) were obtained from Thermo Fisher Scientific. Normal human gastric mucosal epithelial cell lines (GES-1) and GC cell lines (MFC) were acquired from the American Type Culture Collection (Manassas, VA, USA). The cells were incubated in DMEM supplemented with 10% FBS under standard culture conditions (5% CO2, 37 °C).

### 2.6. Cell uptake of PLGA@icaritin

MFC cells were seeded into confocal laser scanning microscope imaging (CLSM) dishes at a density of 1 × 10^5^ cells per well and cultured for 24 h, followed by treatment with PLGA@Icaritin NPs, which were pre-modified with fluorescein isothiocyanate (FITC). After incubation for 0, 2, 4, and 12 h, the cells were washed three times with PBS, fixed with 4% paraformaldehyde, and stained with 4′,6-diamidino-2-phenyllindole (DAPI) for 10 min. The cells were washed twice with PBS, and fluorescence images were captured using CLSM (Olympus, Tokyo, Japan).

Similarly, MFC cells treated with FITC-labeled PLGA@Icaritin NPs were analyzed using flow cytometry. The absorption efficiency of PLGA@Icaritin NPs was analyzed using FlowJo software (TreeStar, Ashland, OR, USA).

### 2.7. Cell counting kit 8 assay

To evaluate the anti-tumor effect of PLGA@Icaritin, MFC cells were seeded into 96-well plates and cultured for 24 h in DMEM supplemented with 10% FBS. The cells were incubated with different concentrations of icaritin, PLGA, and PLGA@Icaritin, and cell viability was analyzed using the Cell Counting Kit-8 assay. This experiment was repeated three times.

### 2.8. Cell cycle distribution

MFC cells were seeded into 6-well plates at 1 × 10^6^ cells per well and cultured for 24 h, followed by treatment with the control (DMEM medium supplemented with 10% FBS), free icaritin, PLGA nanocarriers, or PLGA@Icaritin NPs. The cells were fixed overnight with 70% (w/v) ice-cold ethanol and then analyzed using flow cytometry (BD FACS Caliber™, BD Biosciences, Franklin Lakes, NJ).

### 2.9. Transwell assay

Transwell migration and invasion assays were conducted using a transwell chamber (Corning, Inc., Corning, NY). In the migration assay, 2 × 10^4^ MFC cells suspended in 100 μL serum-free DMEM were seeded into the upper compartment of the chamber, and 800 μL DMEM containing 10% FBS was added to the lower compartment of the chamber. The cells were incubated with PLGA, icaritin, or PLGA@Icaritin for 24 h. The GC cells were fixed with 4% paraformaldehyde for 30 min and stained with 0.1% crystal violet. Non-migrating cells in the upper chamber were carefully removed using a cotton swab. Migrated cells on the lower surface were photographed using an Olympus IX70 inverted microscope in five randomly selected visual fields, and the migrated cells were quantified using Image-Pro Plus 6.0 software (Premier Biosoft, Rockville, MD, USA). Each assay was performed at least three times. For the invasion assay, the upper compartment was precoated with 100 μL of Matrigel. All other processes were the same as those described for the Transwell migration assay.

### 2.10. Mitochondrial oxidative damage

To evaluate the mitochondrial status, MFC cells (1 × 10^6^) were seeded into 6-well plates and incubated with different agents (control, icaritin, PLGA, and PLGA@Icaritin) for 24 h. After washing twice with PBS, MFC cells were collected, incubated with DCFH-DA solution (10 μmol/L) in the dark at 37 °C for 20 min, and analyzed using a flow cytometer (BD FACS Caliber™). The mitochondrial membrane potential was detected using the fluorescent probe JC-1. MFC cells (1 × 10^6^) were treated with icaritin, PLGA, PLGA@Icaritin, or the control for 24 h, and then incubated with 2 μM JC-1 at 37 °C for 20 min and analyzed using a flow cytometer (BD FACS Caliber™). When the MFC cell density reached approximately 70% on the confocal dish, the cells were treated with culture medium containing 10% serum, PLGA, icaritin, or PLGA@Icaritin for 24 h. The cells were washed with PBS twice for 5 min each time, fixed with 4% paraformaldehyde, permeabilized with 0.1% Triton X-100 at room temperature for 15 min, and the incubated with 5% BSA (Cell Signaling Technology, Danvers, MA) at 37 °C for 60 min. The cells were incubated overnight at 4 °C with anti-TOMM20 antibody (1:500; ab186735, Abcam, Cambridge, UK) or 8-OHdG antibody (1: 500; NB600-1508, Novus Biologicals, Littleton, CO, USA). After washing with PBS three times, the MFC cells were incubated with Alexa Fluor 568-conjugated secondary antibody (1:1000; 1793903, Thermo Fisher Scientific) or goat anti-rabbit IgG (Alexa Fluor 488) (ab150077) in the dark for 1 h followed by incubation with DAPI at room temperature for 10 min. Confocal microscopy (Ti A1, Nikon, Tokyo, Japan) was performed to observe the fluorescence of the treated cells.

### 2.11. Detection of ICD biomarkers

Treated MFC cells were collected and fixed with 4% paraformaldehyde for 10 min. After blocking for 1 h at room temperature with 5% BSA, the cells were incubated with rabbit anti-rabbit chaperone CRT or large amounts of HMGB1 primary antibodies at 4 °C overnight. The cells were incubated with phycoerythrin- or FITC-labeled goat anti-rabbit secondary antibodies for an additional 2 h. Finally, the nuclei were stained with DAPI, and the CRT and HMGB1 expression levels were observed using CLSM (Olympus).

### 2.12. *In vivo* anti-tumor effects

To develop the tumor model, MFC cells were harvested and suspended in PBS; 50 μL (at a density of 1 × 10^8^/mL) of this suspension was injected into the right flank of BALB/c mice. When the tumor volumes exceeded 50 mm^3^, the mice were randomly divided into four groups (saline as control group, icaritin, free PLGA, and PLGA@Icaritin-treated group). The dose of icaritin utilized in the mice was 5 mg/kg. The tumor volume and body weight were recorded daily for 28 days, after which the mice were euthanized.

To detect the immune response *in vivo*, tumor tissues were harvested and digested into a single-cell suspension. The cells were labeled with allophycocyanin-anti-CD3, FITC-anti-CD4, and phycoerythrin-anti-CD8 antibodies. After 45 min of incubation, the cells were collected to detect CD3^+^CD4^+^ and CD3^+^CD8^+^ T cells using a flow cytometer following standard protocols.

### 2.13. Enzyme-linked immunosorbent assay

The mice were sacrificed at the end of the experiments, and tumor tissues were collected from all mice. The tumor tissue was suspended in an equal volume of PBS, homogenized, and centrifuged at 12,000 rpm for 10 min. The supernatant was collected to detect interferon (IFN)-γ, tumor necrosis factor (TNF)-α, and interleukin (IL)-6 levels.

### 2.14. Statistical analysis

Data were showed as mean ± standard deviation (SD). All experiments were performed at least three times. In addition, the difference between subgroups were analyzed with one-way ANOVO test. When *p* value <.05, the significant difference was considered.

## 3. Results and discussion

### 3.1. Preparation and characterization of PLGA@icaritin NPs

**Figure 1.**
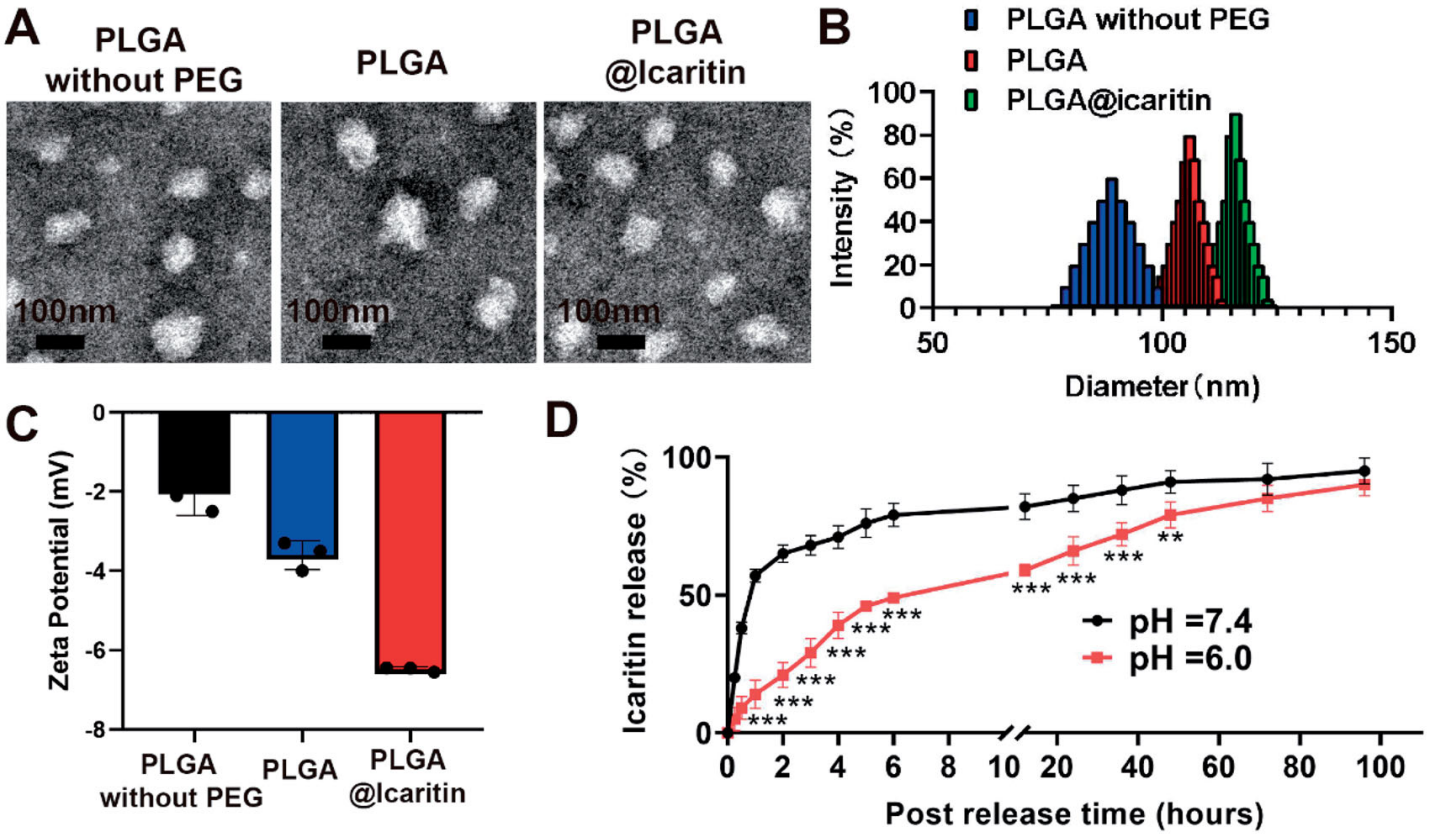
Properties of PLGA@Icaritin. (A) TEM image of PLGA without PEG, PLGA and PLGA@Icaritin. Scale bar, 100 nm. (B) The particle size and surface charge of PLGA without PEG, PLGA and PLGA @Icaritin. (C) The Zate potential of PLGA without PEG, PLGA and PLGA@Icaritin. (D) The release curve of PLGA@Icaritin at pH = 6 and pH = 7.4 solutions. Data are shown as the mean ± SD, *n* = 3. ***Indicates *p* < 0.001.

PLGA is a biodegradable material suitable as a nanodrug carrier because of its biodegradability, small size, and high biocompatibility. However, the mucin permeability of PLGA is hindered by hydrophobic interactions with mucin fibers (Adlravan et al., 2021). PEG has excellent biocompatibility and can significantly improve the stability and prolong the circulation time of NPs in the body. Copolymerization of PLGA with PEG improves the cellular penetration and stability of PLGA. Nanocarriers provide safe and powerful cancer treatment methods by effectively delivering therapeutic agents to targeted tumor tissues (Chen et al., 2021). In PLGA NP loading systems, preferential release of drugs at tumor sites and reduction of off-target cytotoxicity during circulation can be achieved (Zheng et al., 2019), mainly because of the nanometer particle size (10–220 nm) of PLGA (Fasehee et al., 2016). Because of the unique advantages of PLGA drug-loading systems, we designed a nanocarrier loaded with icaritin. PLGA, a self-assembling amphiphilic copolymer, was used to deliver icaritin. Dispersion of prepared NPs was observed and validated using transmission electron microscopy (Figure 1(A)). Figure 1(B) shows that PLGA@Icaritin exhibited a unimodal size distribution. The diameters of PLGA without PEG, PLGA, and PLGA@Icaritin were ∼90, ∼110, and ∼120 nm, respectively. Thus, the loading of the drug only slightly increased the particle size of PLGA. The zeta potentials of PLGA without PEG, PLGA, PLGA@icaritin were approximately −2.5, −4, and −6.8 mV, respectively. These data show that the negative charge on the surface of PLGA@Icaritin NPs was slightly increased, which may improve their circulation stability *in vivo* (Faramarzi et al., 2019) (Figure 1(C)). Similarly, Chen et al. synthesized PLGA-PEG NPs with a particle size of 101.4 ± 2.1 nm, which is similar to our results (Chen et al., 2021). The main objective of using NPs is complete loading of the drug in the carrier, which was evaluated by performing FTIR of PLGA, icaritin, and PLGA@Icaritin, as shown in Figure 1(D). The FTIR spectra of PLGA showed stretching peaks at 1746 and 1081 cm^−1^. The FTIR spectrum of pure icaritin exhibited a strong and wide -OH-stretching peak at 3311 cm^−1^. FTIR spectroscopy of PLGA@Icaritin demonstrated that icaritin was successfully loaded into PLGA (supplemental Figure S1). According to the UV-vis spectra results, icaritin showed a specific absorption band (supplemental Figure S2), which was used to determine the loading efficiency. The encapsulation efficiency and drug loading efficiency were 88.1 ± 1.8% and 1.8 ± 0.06%, respectively. To evaluate the drug release curve, the *in vitro* release of icaritin was monitored in buffers with pH values mimicking those of the blood plasma (pH 7.4) and tumor microenvironment (pH 6.0). Over the 100-h release period, the release rate of the drug significantly accelerated at a mildly acidic pH (6.0) ranging from 0.5 h − 48 h time points, compared to those at pH 7.4 (Figure 1(D)). Taken together, icaritin was successfully loaded into the PEG-modified PLGA NPs.

**Figure 2.**
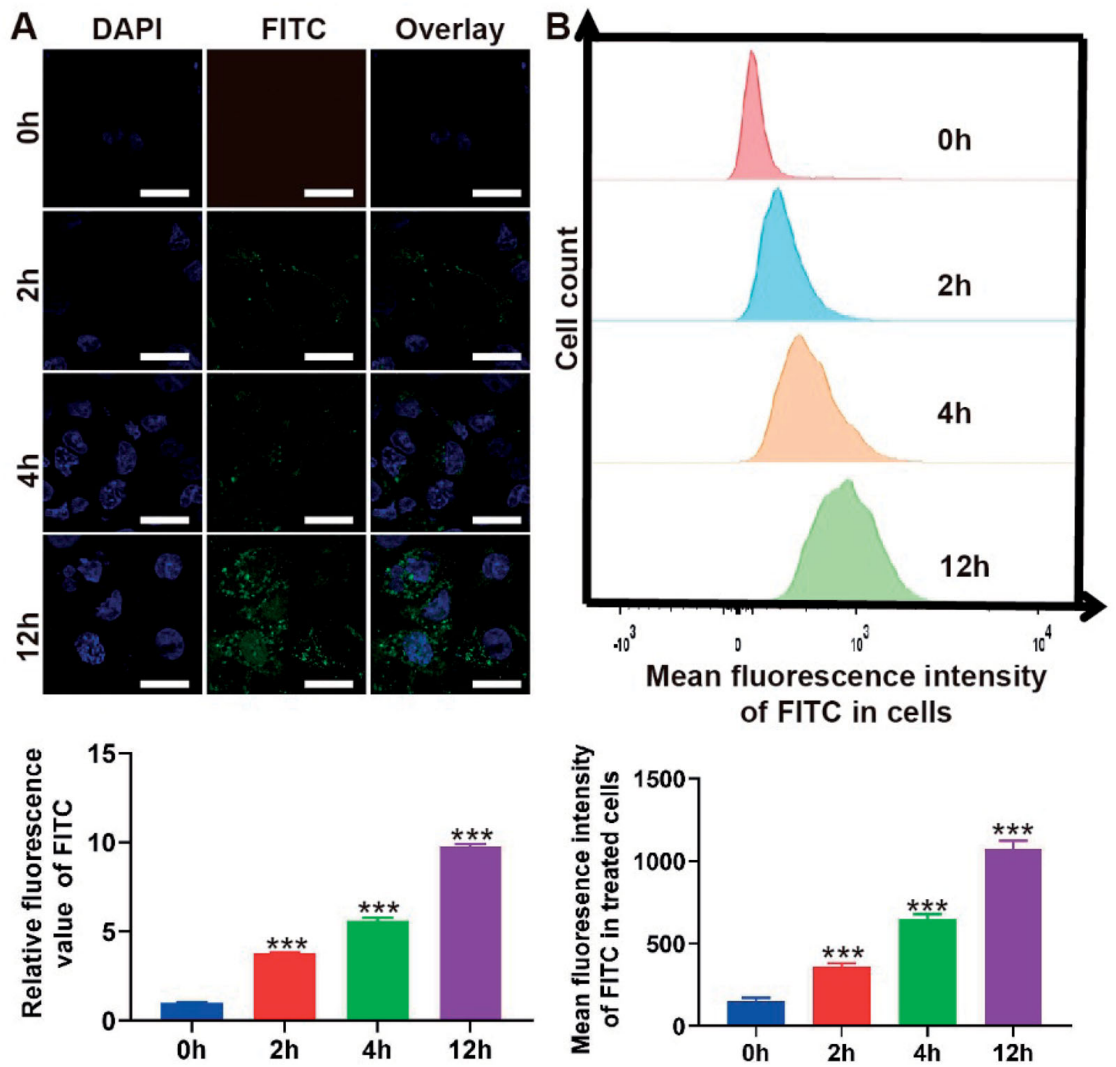
Cell uptake of PLGA@Icaritin. (A) CLSM image of MFC cells treated with PLGA@Icaritin for 0–12 h, and corresponding quantification of CLSM images using the green mean fluorescence intensity of FITC. Scale bar, 20 μm. (B) Flow cytometry images of MFC cells treated with PLGA@Icaritin for 0–12 h, and corresponding quantification of green mean fluorescence intensity of FITC inside cell. Data are shown as the mean ± SD, *n* = 3. ***Indicates *p* < 0.001.

### 3.2. *In vitro* cellular uptake of PLGA@Icaritin NPs

To confirm the uptake of PLGA@Icaritin, MFC cells were co-incubated with FITC-labeled NPs from 0 to 12 h, and the absorption efficiency was analyzed using CLSM and flow cytometry. CLSM images and the corresponding statistical results revealed no green fluorescence at 0 h. At 2 h, significant FITC fluorescence was observed, and the intensities at 4 and 12 h were ∼2- and ∼5-fold higher than those at 2 h, respectively (Figure 2(A)). The flow cytometry results revealed a similar tendency, as negligible green fluorescence was observed at 0 h. Compared to the mean fluorescence intensity at 2 h, that at 4 and 12 h exhibited ∼2- and ∼3-fold changes, respectively (Figure 2(B)). Compared with drugs alone, drugs encapsulated in PLGA showed comparatively higher drug uptake by cancer cells (Tabatabaei et al., 2016). Therefore, both the CLSM and flow cytometry results demonstrated that PLGA@Icaritin efficiently entered GC cells in a time-dependent manner.

### 3.3. Plga@icaritin NPs inhibit GC cell proliferation and metastasis

**Figure 3.**
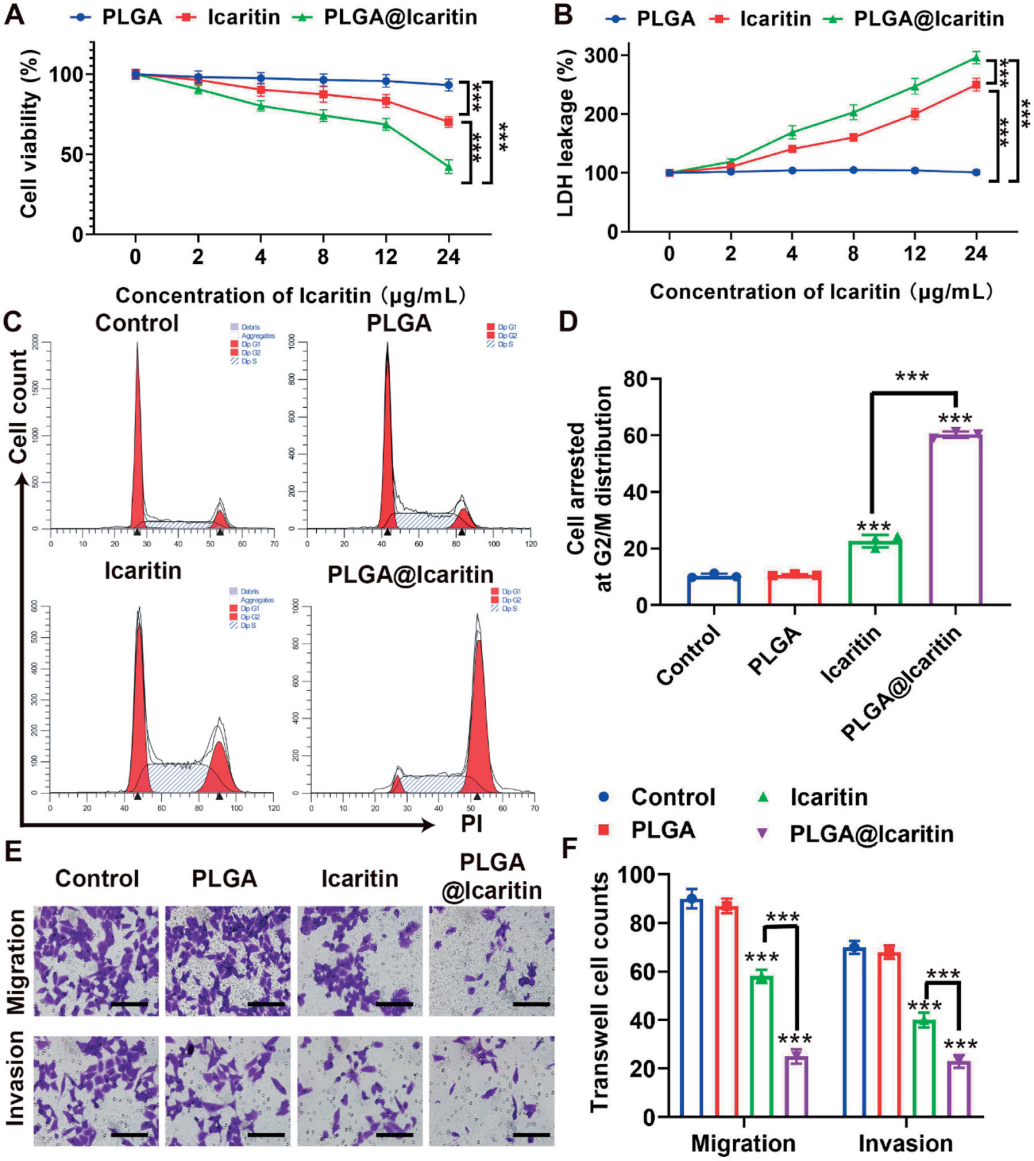
*In vitro* anti-tumor effect of PLGA@Icaritin. (A) Cell Vabilities of MFC cells after incubation with PLGA, icaritin and PLGA@Icaritin. (B) LDH leakage of MFC cells after incubation with PLGA, icaritin and PLGA@Icaritin. (C) Cell cycle distribution images. (D) Statistical graph of G2/M phase rates. (E,F) The effect of PLGA, icaritin and PLGA@Icaritin on migration and invasion was assessed with transwell migration assay and tranwell invasion assay, respectively. 200×. Data are shown as the mean ± SD, *n* = 3. ***Indicates *p* < 0.001.

Effectively treating GC remains difficult, mainly because of emerging multidrug resistance. Epimedium was first described in Shennong’s herbal classic and is increasingly being used to prevent and treat cardiovascular, skeletal, neuroendocrine, immune, and other diseases (Lv et al., 2017). Icaritin is a hydrolysate of icariin, one of the main active components of Epimedium. In recent years, icaritin has been considered as a potential cancer suppressor because of its inhibitory effect on tumors such as lymphoma, lung cancer, and prostate cancer (Wu et al., 2015, 2020; Zhao et al., 2020). Wu et al. showed that icaritin inhibits cell proliferation and induces apoptosis in lymphoma. Zhao et al. demonstrated that icaritin inhibits the proliferation of lung cancer cells. Similarly, icaritin inhibited the proliferation of bladder cancer cells. However, icaritin has limitations such as unclear targeting and poor bioavailability. To address this issue, we loaded icaritin into a nanosized drug delivery system (PLGA@Icaritin), and its anti-tumor effect on GC was investigated. MFC cells were incubated with free icaritin, PLGA, and PLGA@Icaritin NPs at different concentrations, and cell proliferation was analyzed in a Cell Counting Kit-8 assay. The results showed that PLGA did not induce significant cell death at any of the tested concentrations, as all groups showed >90% cell viability. MFC cells treated with increasing concentrations of icaritin showed a dose-dependent decrease in viability and increased lactate dehydrogenase leakage (Figure 3(A,B)). PLGA@Icaritin (24 μg/mL of icaritin) induces lower cell viability rates (42.3%) compared to free icaritin (70.2%) and causes higher lactate dehydrogenase leakage (∼300%) than free icaritin (∼250%), demonstrating that the anti-tumor effect of PLGA@Icaritin on MFC cells was much greater than that of free icaritin (Figure 3(A,B)). To further examine the anti-proliferative effect of PLGA@Icaritin, we determined the cell cycle distribution of MFC cells by flow cytometry. More G2/M cells were arrested in the PLGA@Icaritin group (∼50%) than in the PLGA group (∼10%) and icaritin group (∼25%) (Figure 3(C,D)). Thus, PLGA@icaritin showed better anti-growth potential by arresting more GC cells at G2/M phase. Transwell assays revealed that PLGA@Icaritin (∼50%) attenuated the migratory and invasive abilities of the cells *in vitro* compared to the effects of icaritin (∼25%), whereas PLGA did not influence GC cell metastasis compared to that in the control (Figure 3(E,F)). Cell growth and the migratory and invasive abilities were significantly different. In summary, compared to free icaritin, PLGA@Icaritin exerted a more powerful inhibitory effect based on the significant differences in cell growth, migration, and invasion abilities induced by the NPs. However, the anti-tumor mechanism of PLGA@Icaritin remains unclear.

### 3.4. Plga@icaritin NPs induce mitochondrial oxidative damage in GC cells

Mitochondria provide energy to cells and participate in the basic functions of cells, including ATP production, intracellular Ca^2+^ regulation, reactive oxygen species (ROS) production and clearance, regulation of apoptotic cell death, and activation of proteases (Martin-Fernandez & Gredilla, 2018). Mitochondrial dysfunction and oxidative stress are mainly related to aging, cancer, age-related neurodegeneration, and metabolic syndrome (Ippolito et al., 2020). When cells are stimulated by certain external factors, mitochondrial electron transport is blocked and matrix metalloproteinases (MMPs) are greatly decreased (Guo et al., 2020; Chen et al., 2021). The loss of MMPs mediates the generation of ROS, which induce apoptosis and inhibits tumor growth (Montemurro et al., 2019). Mitochondria are central organelles for the survival of cancer cells. Icaritin can induce mitochondrial damage-related apoptosis, accompanied by a significant decrease in MMPs and the generation of large amounts of ROS (Li et al., 2017). Disturbance of the oxidation-antioxidant balance caused by increased ROS production or decreased ROS-scavenging ability can lead to many diseases, including cancer. Interestingly, icaritin was also reported to induce mitochondrial fission and fragmentation (Yu et al., 2020). Therefore, we analyzed the characteristics of mitochondrial damage in PLGA@Icaritin-treated GC cells, such as mitochondrial-related markers (ROS and MMPs).

**Figure 4.**
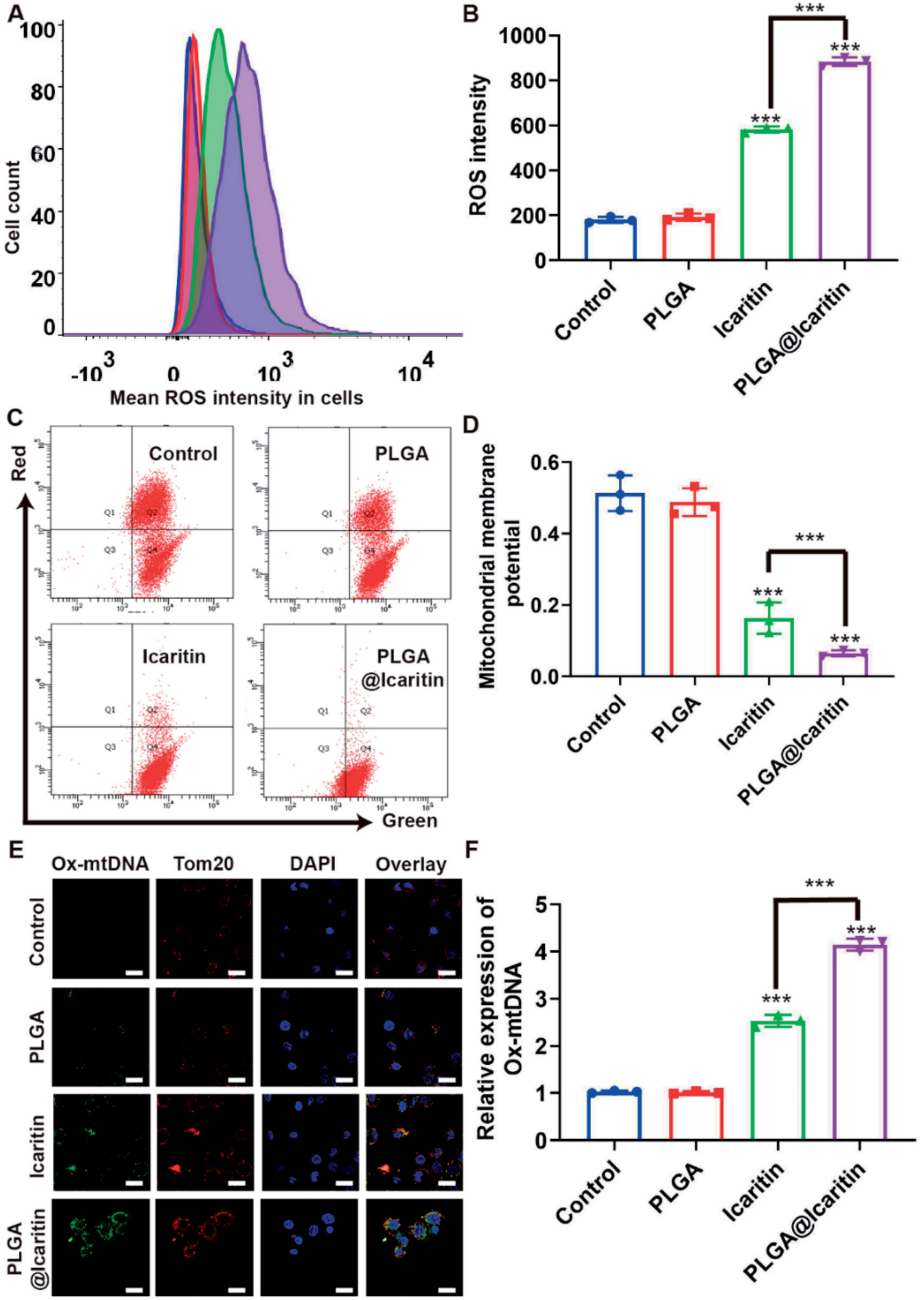
Mitochondrial oxidative damage induced by PLGA@Icaritin. (A) The generation of ROS in MFC cells treated with PLGA, icaritin and PLGA@Icaritin. (B) Corresponding quantification of green mean fluorescence intensity of FITC inside cells. (C,D) Flow cytometric analysis of untreated, PLGA-treated, icaritin-treated and PLGA@Icaritin-treated MFC cells stained with JC-1 after 72 h of exposure for mitochondrial transmembrane potential (MMP) evaluation. (E,F) Ox-mtDNA and TOM20 images of MFC cells, detected by CLSM. Scale bar, 20 μm. Data are shown as the mean ± SD, *n* = 3. ***Indicates *p* < 0.001.

The flow cytometry results showed that ROS generation in MFC cells treated with PLGA@Icaritin was nearly two-fold higher than that in the free icaritin groups (Figure 4(A,B)). We then tested the mitochondrial membrane potential of MFC cells using the JC-1 assay. The concentration of JC-1 in the mitochondria of normal cells increases and forms polymers that emit strong red fluorescence, which can be detected using the FL-2 channel of flow cytometry. As shown in Figure 4(C,D), the ratio of red and green fluorescence in the PLGA@Icaritin group was ∼10-fold lower than that in control group, indicating that MMPs of MFC cells treated with PLGA@Icaritin were significantly reduced compared to other groups. These results indicate that P PLGA@Icaritin can induce apoptosis of MFC cells by mediating mitochondrial damage.

Large amounts of ROS not only attack mitochondria but also cause mtDNA mutations. Tom20 is a downstream factor of ROS that can sense ROS signal transduction and transmit the signal to the mitochondria to induce CytoC release, thus activating the apoptosis pathway (Lv et al., 2021). Considering these findings, we examined the colocalization of Ox-mtDNA and Tom20. Compared with the control group, the expression of Ox-mtDNA in MFC cells treated with free icaritin was increased by more than two-fold, whereas that in the PLGA@Icaritin group was 2-fold that in the free icaritin group (Figure 4(E,F)). Therefore, the anti-tumor effect of PLGA@Icaritin may be related to apoptosis caused by mitochondrial oxidative damage, including damage to mitochondrial DNA.

### 3.5. Plga@Icaritin induce immunogenic cell death *in vitro*

**Figure 5.**
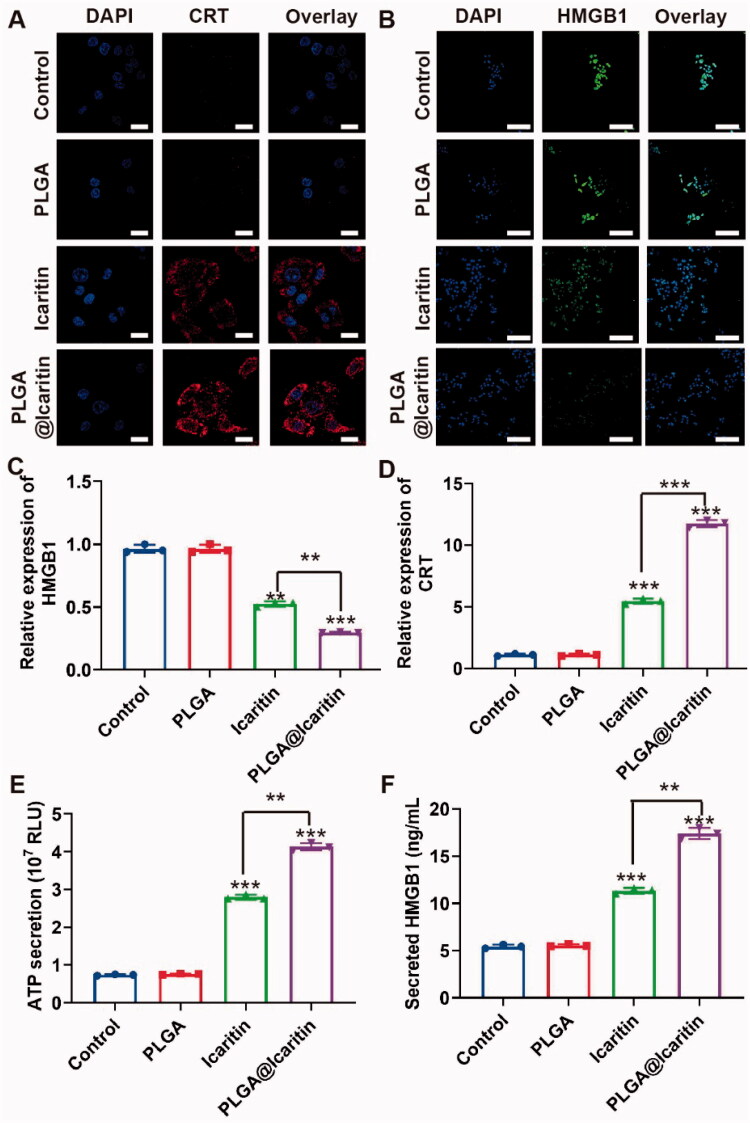
ICD induced by PLGA@Icaritin *in vitro*. (A) CRT image of MFC cells after the incubation with PLGA, icaritin and PLGA@Icaritin. Scale bar, 30 μm. (B) HMGB1 image of MFC cells after the incubation with PLGA, icaritin and PLGA@Icaritin. Scale bar, 30 μm. (C) Relative expression of CRT in MFC cells after the incubation with PLGA, icaritin and PLGA@Icaritin. (D) Relative expression of HMGB1 in MFC cells after the incubation with PLGA, icaritin and PLGA@Icaritin. (E) The ATP secretion of MFC cell after incubation with PLGA, icaritin and PLGA@Icaritin. (F) Secreted HMGB1 of MFC cell after incubation with PLGA, icaritin and PLGA@Icaritin. Data are shown as the mean ± SD, *n* = 3. **Indicates *p* < 0.01; ***indicates *p* < 0.001.

ICD is a process in which tumor cells die from non-immunogenicity to immunogenicity (Hou et al., 2020). Tumor cells that undergo immunogenic cell death release related molecules such as CRT, HMGB1, and ATP, which stimulate an anti-tumor response (Salas-Benito et al., 2021). Yu et al. demonstrated that icaritin induces ICD in cancer cells by inducing mitophagy (Li et al., 2019; Yu et al., 2020). Several recent studies reported that mitochondrial damage is closely associated with the occurrence of ICD. Chao et al. first reported that mitochondrial oxidative stress can cause abandonment and large-scale ICD, which correlates with important organelles with immunogenic death (Chen et al., 2019). Furthermore, Bianca et al. found that the release of mitochondrial DNA activates the inflammasome for efficient IL-1β secretion that causes ICD (Li et al., 2019), demonstrating that oxidative damage to mtDNA is an important cause of ICD. Combined with our findings, our NPs may also cause ICD by inducing mitochondrial damage. To investigate whether PLGA@Icaritin induces ICD in GC cells, we evaluated the expression levels of CRT and HMGB1 in MFC cells treated with PLGA@Icaritin. The CLSM and flow cytometry results showed that the expression levels of CRT and HMGB1 in MFC cells treated with PLGA@icaritin group were enhanced compared with those in the PLGA group and icaritin group; cells treated with PLGA@Icaritin showed brighter red or green fluorescence for CRT and HMGB1, respectively (Figure 5(A–D)). The ATP secretion of MFC cells treated with PLGA@Icaritin was increased (Figure 5(E)), indicating greater more ICD effects and revealing the potential for combining these NPs with tumor immunotherapy.

### 3.6. Intravenous PLGA@Icaritin administration effectively inhibits tumor growth without eliciting systemic toxic side effects

**Figure 6.**
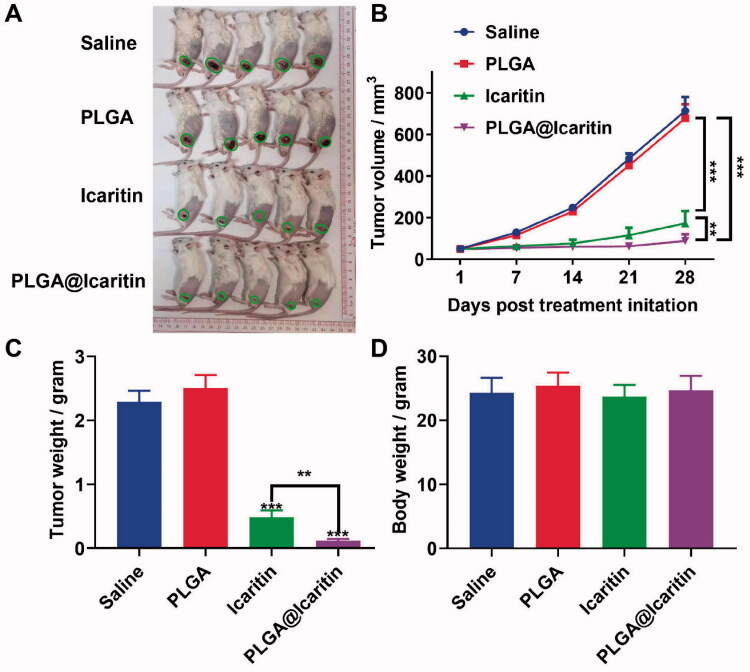
*In vivo* anti-tumor effect of PLGA@Icaritin. (A) Image of tumors derived from mice treated with saline, PLGA, icaritin and PLGA@Icaritin. (B) Tumor volume of MFC tumors isolated from mice. (C) Tumor weight of MFC tumors isolated from mice. (D) Body weight of MFC tumor-bearing mice. Data are shown as the mean ± SD, *n* = 3. **Indicates *p* < 0.01; ***indicates *p* < 0.001.

We confirmed that PLGA@Icaritin can effectively exert anti-tumor proliferation effects *in vitro* and tested the *in vivo* targeting capability and tissue distribution profiles of PLGA@icaritin NPs pre-incubated with Cy7. Tumor-bearing mice were treated with PLGA@Icaritin-Cy7 via tail vein injection. After 2, 4, and 6 h, the mice were imaged using a small-animal *in vivo* fluorescence imaging system. The *in vivo* fluorescence images indicated that PLGA@Icaritin accessed the cancer region in 2 h, and then gradually became enriched at the tumor site from 4 to 6 h (supplemental Figure S3A). Tumor-bearing mice were sacrificed at 6 h post-injection, and major organs were collected for fluorescence imaging. *Ex vivo* imaging showed that PLGA@Icaritin NPs accumulated in the cancer region (supplemental Figure S3B), confirming their passive tumor-targeting abilities through enhanced permeability and retention (EPR) effects. Next, we investigated the anti-cancer effect of PLGA@Icaritin on MFC-tumor-bearing BALB/c mice via vein injection every three days. The results of *in vivo* experiments revealed that the tumor volume and weight in the saline group and PLGA group did not change significantly, showing that these agents did not inhibit GC cell proliferation, whereas PLGA@Icaritin suppressed tumor growth by almost 80% compared with that in the saline group; the inhibition rate was higher than the ∼50% inhibition achieved by free icaritin (Figure 6(A)). PLGA@Icaritin showed the same inhibitory effect on tumor weight as on tumor volume (Figure 6(B,C)), indicating that PLGA@icaritin suppressed tumorigenesis *in vivo*. Further studies are required to establish the safety of this drug for *in vivo* use. To further examine the potential side effects of PLGA@Icaritin, we tested the weight change of mice throughout the experiment; the results showed that changes in the weight of mice in the four groups did not significantly differ (Figure 6(D)), indicating that PLGA@Icaritin had no significant side effects. Fatemah et al. also found that drugs encapsulated in PLGA were more effective than free drugs for inhibiting tumor growth (Tabatabaei et al., 2016), indicating their potential for treating GC.

### 3.7. Anti-tumor immunity of PLGA@Icaritin NPs

**Figure 7.**
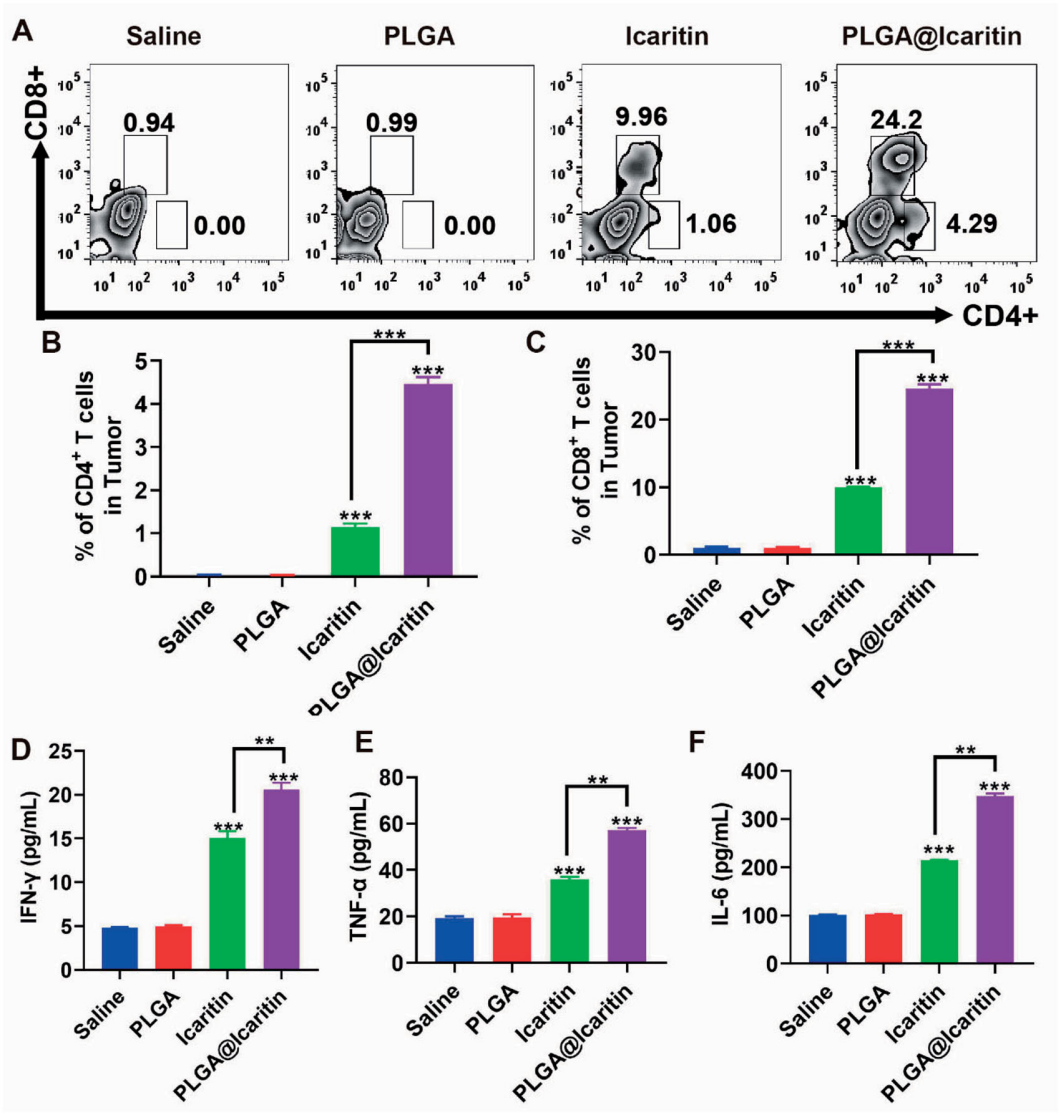
Immune response induced by PLGA@Icaritin. (A) Production of CD3 + CD4+ and CD3 + CD8+ T cells in splenic lymphocytes harvested from immunized mice. (B,C) The proportions of CD3 + CD4+ and CD3 + CD8+ T cells in splenic lymphocytes. (D) Quantitative analysis of IFN-γ in serum samples of MFC tumor-bearing mice after different treatments. (E) Quantitative analysis of TNF-α in serum samples of MFC tumor-bearing mice after different treatments. (F) Quantitative analysis of IL-6 in serum samples of MFC tumor-bearing mice after different treatments. Data are shown as the mean ± SD, *n* = 3. **Indicates *p* < 0.01; ***indicates *p* < 0.001.

After the release of HMG1, activated effector T cells penetrate the tumor location and kill cancer cells through the cognitive antigen combined with the T cell receptor and major histocompatibility complex class I. Dying cancer cells release additional tumor-associated antigens to increase the breadth and depth of the immune response (Kroemer et al., 2013). Compared to traditional methods, nanotechnology provides an opportunity to effectively deliver optimal doses of ICD inducers to specific tissues or cell types, enhance their effectiveness, and reduce their side effects (Duan et al., 2019). We demonstrated that PLGA@Icaritin NPs induced ICD *in vitro*; thus, we explored their antitumor immune response *in vivo*. Tumor tissues were harvested post-treatment to analyze the associated immune signals using enzyme-linked immunosorbent assay. The tumor infiltration of T cells after treatment was measured. The results showed that the icaritin and PLGA@Icaritin groups both recruited more CD3CD4 and CD3CD8 T cells to tumor tissues compared to the physiological saline treatment group (Figure 7(A,B)). Furthermore, immune cytokines were analyzed to evaluate their immunotherapeutic effects. As shown in Figure 7(D–F), secretion of IFN-γ, TNF-α, and IL-6 was clearly increased in the icaritin and PLGA@Icaritin groups. Particularly, the secretion of IFN-γ, TNF-α, and IL-6 treated with PLGA@Iicaritin was ∼3-, ∼3.5-, and ∼4-fold higher than that in the saline group. The high infiltration of immune cells into tumor tissues is essential for effective immunotherapy (Qiu et al., 2022). Pro-inflammatory cytokines play important roles in coordinating cell-mediated immune responses and regulating the immune system (Zhang et al., 2021). TNF-α can promote the expression of major histocompatibility complex class I and intercellular adhesion molecules, cooperate with IFN-γ to induce the expression of major histocompatibility complex class II, initiate T cell-mediated cellular immunity, and enhance the cellular activity of T cells, natural killer cells, lymphokine-activated killer cells, and tumor-infiltrating lymphocytes (Jewett et al., 2020). TNF-α promotes DC precursor cells to differentiate into mature DCs with specific genotypes, which can promote autologous T lymphocyte proliferation, activate unsensitized T lymphocytes into tumor-specific cytotoxic T lymphocytes, and induce effective anti-tumor immunity (Eastman et al., ^++++^^2019^; Bent et al., 2021). The higher levels of TNF-α, IFN-γ, and IL-6 in tumor tissue indicated that PLGA@Icaritin can induce natural killer cell proliferation and stimulate the production of TNF-α, IFN-γ, and IL-6. Therefore, PLGA@Icaritin can reshape the tumor immune microenvironment and improve the therapeutic effect of immuno-oncology agents.

## 4. Conclusion

**Scheme 1.**
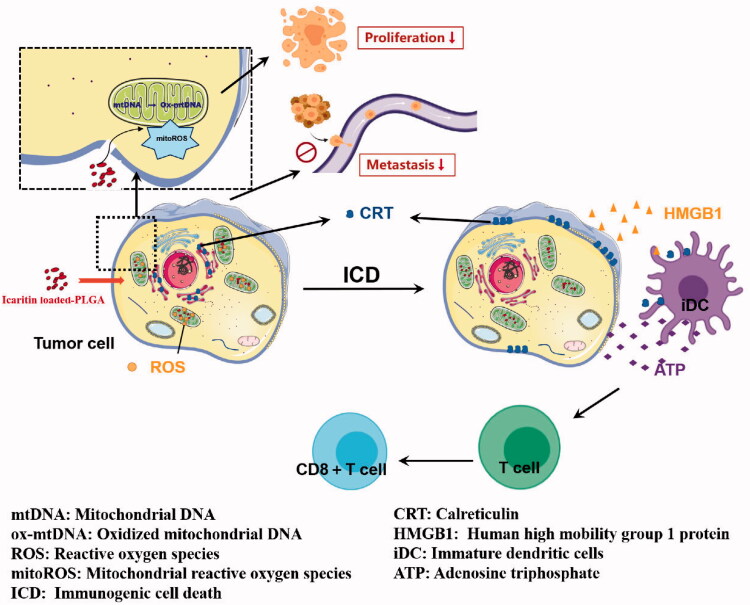
Schematic diagram of the proposed therapeutic mechanisms of PLGA@Icaritin NPs.

We constructed PLGA@Icaritin NPs that showed a good safety profile and effectively inhibited the proliferation and metastasis of GC cells. NPs reduced the tumor viability and migration rates by approximately 30%. Additionally, PLGA@Iicaritin NPs can cause tumor cells to release ROS to attack mitochondria, cause mitochondrial damage and the release of oxidative DNA, induce ICD of tumor cells, and further trigger immune anti-tumor effects. Thus, icaritin may be useful for treating cancer by activating the immune system to cause a sustained anti-tumor effect (Scheme 1).

## Supplementary Material

Supplemental Material
