# Supplementary material for: Icaritin-loaded PLGA nanoparticles activate immunogenic cell death and facilitate tumor recruitment in mice with gastric cancer
Source: Drug Deliv. 2022 May 30;29(1):1712–25. doi: 10.1080/10717544.2022.2079769 (PMC9176696; doi:10.1080/10717544.2022.2079769)
Supplement: Supplemental Material [file IDRD_A_2079769_SM4165.docx]

**Supplementary information**


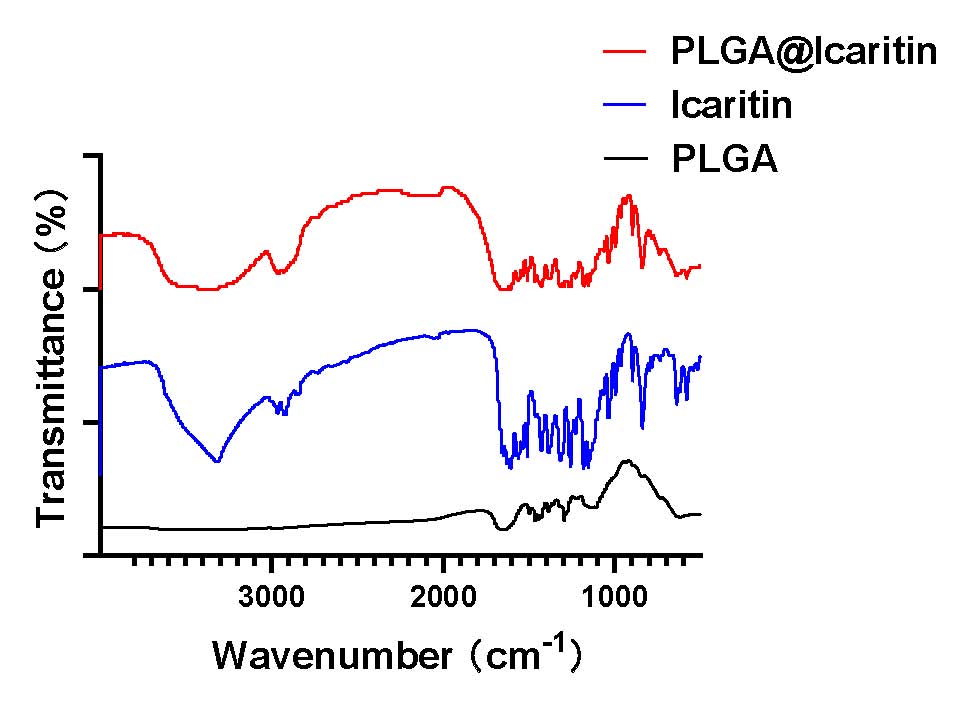


Fig S1. FT IR spectra of PLGA@Icaritin, icaritin and PLGA.


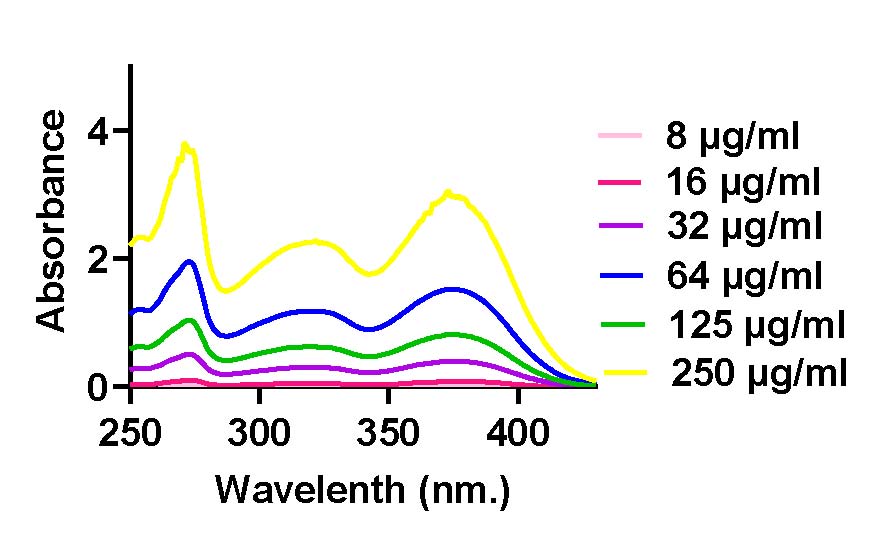


Fig S2. UV-vis spectra of icaritin.


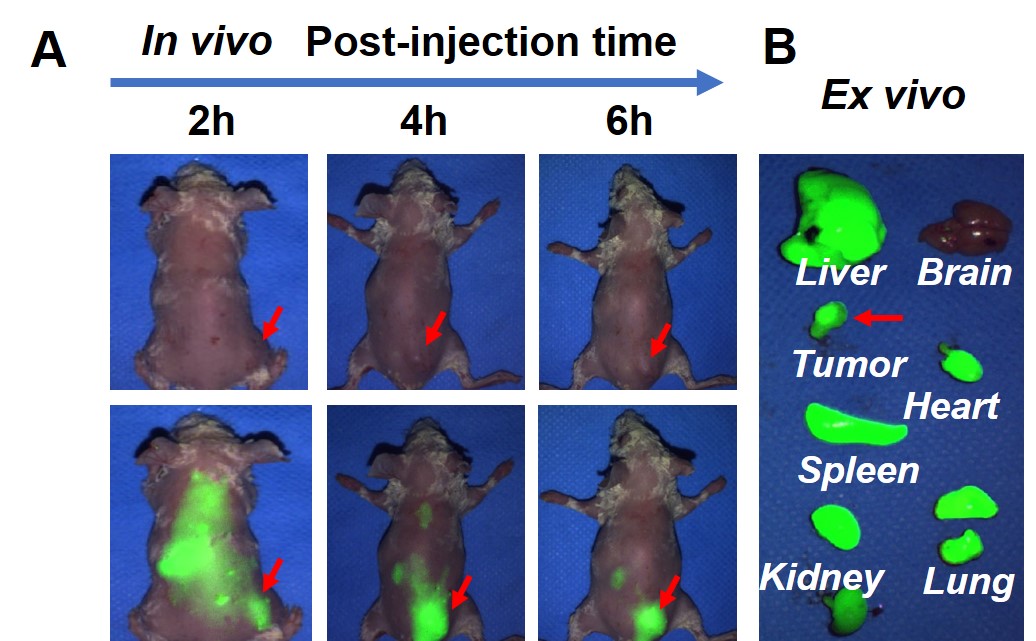


Fig S3. *In vivo* distribution of PLGA@Icaritin NPs.
